# Local temporal Rac1-GTP nadirs and peaks restrict cell protrusions and retractions

**DOI:** 10.1126/sciadv.abl3667

**Published:** 2022-03-23

**Authors:** Jianjiang Hu, Xiaowei Gong, Staffan Strömblad

**Affiliations:** Department of Biosciences and Nutrition, Karolinska Institutet, SE-141 83 Huddinge, Sweden.

## Abstract

Cells probe their microenvironment using membrane protrusion-retraction cycles. Spatiotemporal coordination of Rac1 and RhoA GTP-binding activities initiates and reinforces protrusions and retractions, but the control of their finite lifetime remains unclear. We examined the relations of Rac1 and RhoA GTP-binding levels to key protrusion and retraction events, as well as to cell-ECM traction forces at physiologically relevant ECM stiffness. High RhoA-GTP preceded retractions and Rac1-GTP elevation before protrusions. Notable temporal Rac1-GTP nadirs and peaks occurred at the maximal edge velocity of local membrane protrusions and retractions, respectively, followed by declined edge velocity. Moreover, altered local Rac1-GTP consistently preceded similarly altered traction force. Local optogenetic Rac1-GTP perturbations defined a function of Rac1 in restricting protrusions and retractions and in promoting local traction force. Together, we show that Rac1 plays a fundamental role in restricting the size and durability of protrusions and retractions, plausibly in part through controlling traction forces.

## Teaser

Rac1 plays a fundamental role in restricting the size and durability of cell membrane protrusions and retractions.

## INTRODUCTION

Cell membrane protrusions and retractions are highly dynamic membrane structures involved in cell–extracellular matrix (ECM) interactions, cell migration, and invasion (*1*–*3*). Local dynamic cell membrane protrusion-retraction cycles provide cells with probing, exploratory ability of the surrounding microenvironment (*4*–*6*). The Rho family small guanosine triphosphatases (GTPases) RhoA and Rac1 cycles between guanosine 5′-triphosphate (GTP)–bound and guanosine diphosphate (GDP)–bound states and are central regulators of cytoskeletal dynamics, cell-ECM adhesions, and myosin contraction (*7*–*10*). Coordination of their GTP-bound activities governs protrusion and retraction initiation and reinforcement (*11*, *12*). During membrane protrusion of mammalian cells on glass, RhoA activity and recruitment of the RhoA effector mDia (Diaphanous-related formin) precedes Rac1 and Cdc42 activation (*11*–*14*). Rac1-GTP promotes local cell-ECM adhesion and actin filament assembly that drive cellular protrusions (*7*, *8*, *15*), while RhoA-GTP promotes cell-ECM adhesion maturation, stimulates actin polymerization at the front of the lamellipodia (*16*), and enhances interactions between myosin and actin filaments to drive local contractility. This way, RhoA promotes the initiation and reinforcement of both protrusions and retractions (*17*–*19*). Thus, Rac1-GTP and RhoA-GTP produce cell shape changes with force application at cell-ECM adhesions (*8*, *16*, *17*, *20*, *21*). Local protrusive and contractile forces, in turn, redistribute or reorganize actin filaments, adhesions, and their regulators (*22*–*24*). However, it remains unclear how initiated and reinforced protrusions and retractions may be restrained to facilitate the naturally occurring dynamic cycles of local protrusions and retractions and whether this restrain might involve local alterations of cell-ECM forces.

## RESULTS

**Fig. 1.**
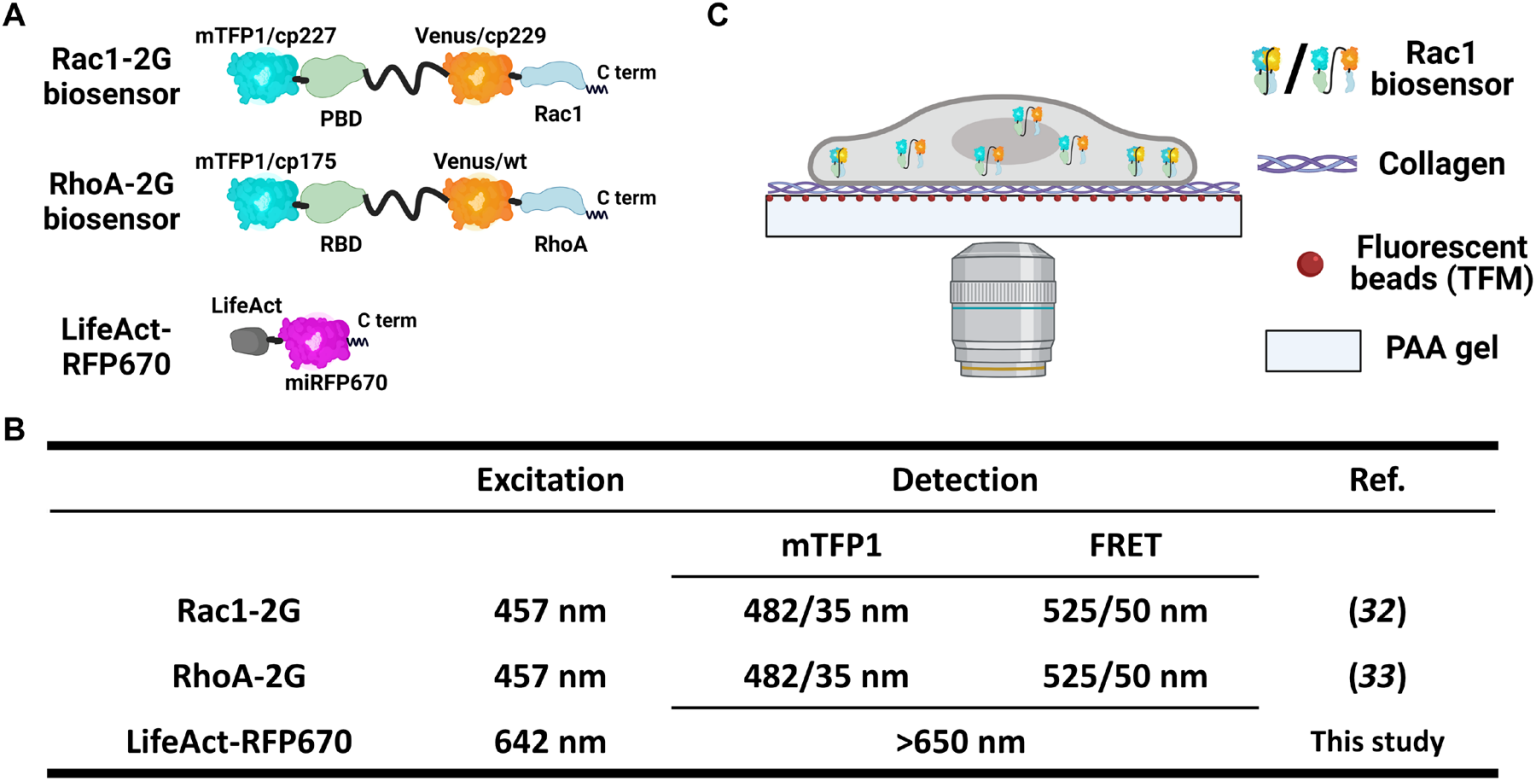
Schematic experimental setup. (**A** and **B**) Genetically encoded fluorescent probes used in this study. (A) Schematic structures of Rac1-2G, RhoA-2G, and LifeAct-RFP670 biosensors. (B) The table shows the excitation and detection settings of the fluorescent probes used in this study. (**C**) Schematic image of the platform for simultaneous measurement of highly resolved GTP-binding activity, cell traction force, and cell membrane movements. HT1080 human fibrosarcoma cells stably expressing genetically encoded FRET biosensors were used to capture Rac1-GTP or RhoA-GTP levels. Cells were seeded onto a collagen type I–coated surface with a physiologically relevant stiffness (Young’s modulus, 6.9 kPa) also surface-labeled with red fluorescent beads to accommodate traction force measurements. Signals were captured via the objective at the bottom providing submicrometer resolution. TFM, traction force microscopy.

The mechanics of the cellular microenvironment, including the substrate stiffness, affects cell membrane behavior and signaling (*23*–*26*). Because conventional glass/plastic surfaces are in the million-fold range stiffer (1 to 7 GPa) than mammalian tissues (*27*), we provided HT1080 human fibrosarcoma cells with collagen type I of physiologically relevant stiffness coated onto a polyacrylamide (PAA) gel (6.9 kPa) (*28*, *29*) also surface-labeled with red fluorescent beads to accommodate traction force measurements (*30*, *31*). Genetically encoded fluorescence resonance energy transfer (FRET)–based biosensors were used to capture Rac1-GTP or RhoA-GTP levels and the membrane protrusion and retraction dynamics (Fig. 1) (*16*, *32*, *33*). Three-dimensional (3D) FRET imaging with lattice light sheet microscopy revealed that the vast majority of the Rac1-GTP and RhoA-GTP were located at the cell-ECM interface (figs. S1 and S2). At membrane ruffles, we observed limited Rac1-GTP and high RhoA-GTP levels, the latter as previously suggested (figs. S3 and S4) (*16*, *33*). We then used conventional confocal microscopy to simultaneously capture Rho GTPase activities and traction forces at submicrometer resolution with 10-s time interval near the cell-ECM interface.

**Fig. 2.**
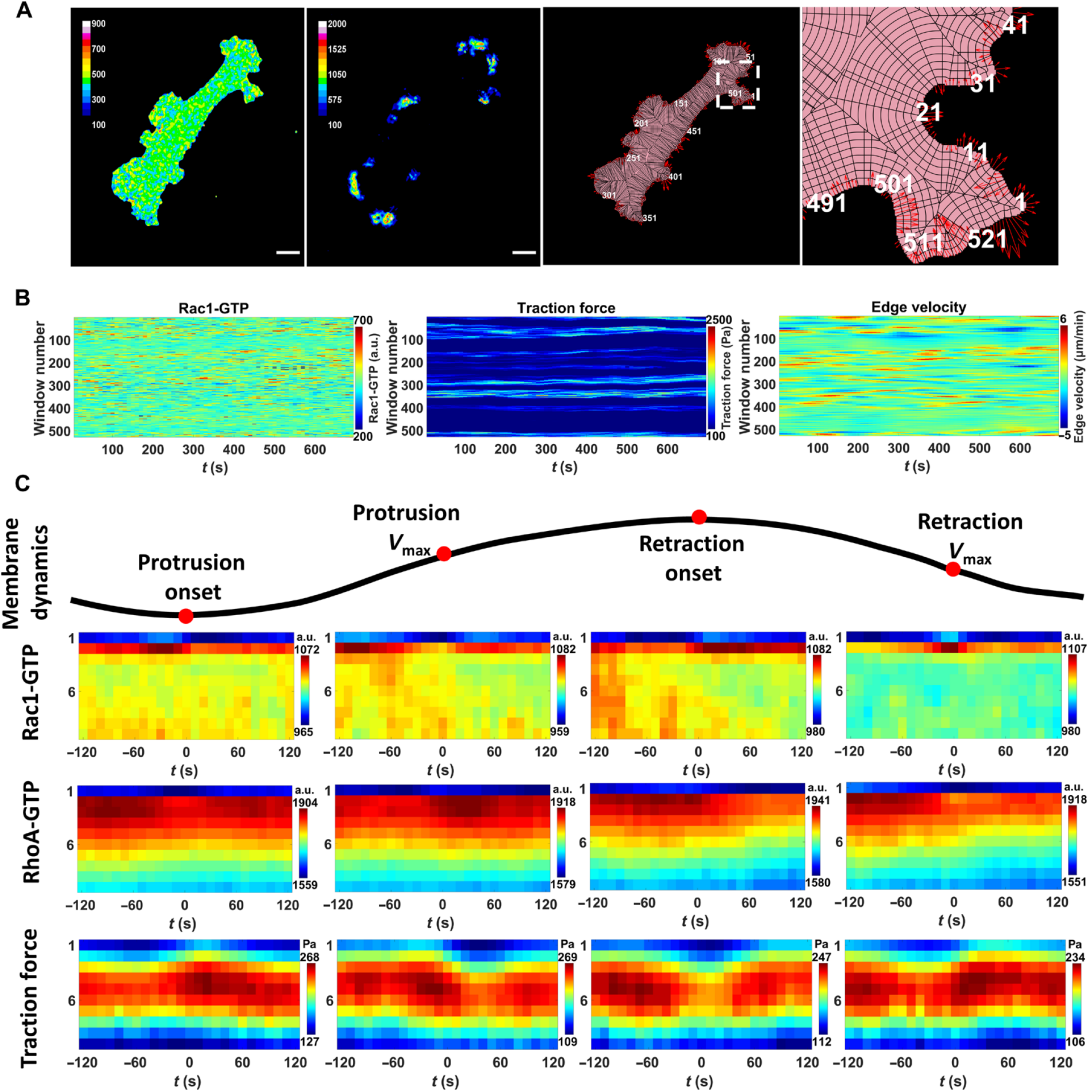
Spatiotemporal analysis of Rac1-GTP, RhoA-GTP, and traction forces at key cell membrane events. (**A**) Sample images of GTP-bound activity, traction force, and cell membrane movements. The sample images show the acquired Rac1-GTP level (left), traction force (middle left), outlay of the window sampling (middle right), and zoom-in of the window sampling (right). Rac1-GTP [arbitrary units (a.u.)] and traction force (pascal) levels are shown in pseudo-colors (inset scales). Window sampling images: pink, segmented cell area; black lines, cell sampling into 1-μm-wide cell edge windows based on local geometry. White, window numbering along the cell edge. Red arrows, instant edge sector velocity indicating cell membrane movement direction and velocity (length of arrows). Scale bar, 20 μm. (**B**) Temporal GTPase activity, traction force, and cell edge velocity. Registered and smoothed GTP-bound activity (left) and traction force (middle) levels in the second windows (1 to 2 μm from the cell edge) all around the cell displayed over time as kymographs, with the corresponding edge velocity (right). Pseudo-colors according to the indicated scales. (**C**) Rac1-GTP, RhoA-GTP, and traction force levels around key cell membrane events. The measurements in segmented cell membrane edge sectors (1 μm wide) aligned to cell membrane protrusion onset, maximal protrusion velocity (protrusion *V*_max_), retraction onset, and maximal retraction velocity (retraction *V*_max_). Rac1-GTP, RhoA-GTP, and traction force mean levels at different depths of the cell (1 to 10 μm; *y* axis) around the specific time points (−120 to +120 s; *x* axis) shown in pseudo-colors. Sample size: Rac1-GTP and traction force, 5852 protrusions and 3817 retractions from 13 cells of two experiments; RhoA-GTP, 8732 protrusions and 4244 retractions from 23 cells of four experiments.

To analyze our time-lapse images, we applied the window sampling method developed by the Danuser Lab (*11*, *13*) to segment the entire cell into 1 μm by 1 μm windows that were tracked over time. This derived time series of local membrane edge dynamics and the corresponding Rac1-GTP/RhoA-GTP and traction force levels in each window from the cell edge to the cell center (Fig. 2, A and B, and movie S1). By aligning and averaging thousands of time series, we obtained a quantitative measure of the relation between the highly localized protrusion/retraction membrane dynamics and the dynamic alterations of Rac1-GTP, RhoA-GTP, and traction force levels.

**Fig. 3.**
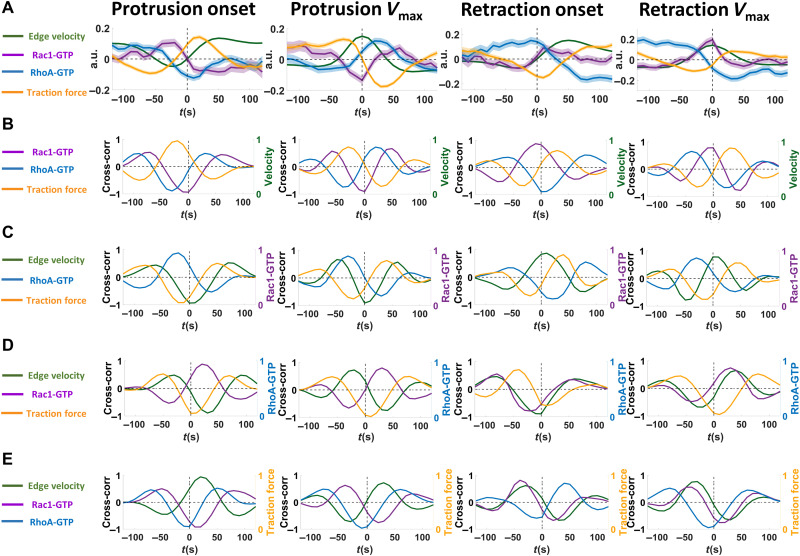
Localized temporal Rac1-GTP nadirs and peaks at the cell membrane maximum velocity of protrusions and retractions. (**A**) Rac1-GTP, RhoA-GTP, and traction force levels around key cell membrane events. The mean values of Rac1-GTP, RhoA-GTP, and traction force levels at the second window (1 to 2 μm from the segmented cell edge) before and after the protrusion onset, protrusion *V*_max_, retraction onset, and retraction *V*_max_, extracted from the data represented in Fig. 2C. The raw data were normalized with the *z* score method before calculating the mean values. Solid lines show the mean values, and the shadows show the 95% confidence intervals. Sample size: Rac1-GTP (velocity and traction force), 5852 protrusions and 3817 retractions from 13 cells of two experiments; RhoA-GTP, 8732 protrusions and 4244 retractions from 23 cells of four experiments. (**B** to **E**) Cross-correlation analyses between cell edge velocity, Rac1-GTP, RhoA-GTP, and traction force. Cross-correlation analysis of mean Rac1-GTP (purple), RhoA-GTP (blue), and traction force (orange) levels in the second window (1 to 2 μm from the segmented cell edge) and corresponding cell edge sector mean velocity (green) toward each other around protrusion onset, protrusion *V*_max_, retraction onset, and retraction *V*_max_. The cross-correlation analysis is centered on (B) cell edge velocity, (C) Rac1-GTP levels, (D) RhoA-GTP levels, and (E) traction force levels. Cross-correlation results are shown in colored lines as indicated on the left side of each graph.

We aligned the time series of all windows from the different cells according to four different edge motion events (*11*, *13*): protrusion onset, maximal protrusion velocity (protrusion *V*_max_), retraction onset, and maximal retraction velocity (retraction *V*_max_). This allowed us to quantify the dynamics of the mean Rac1-GTP, RhoA-GTP, and traction force levels at different depth layers of the cell at the time periods surrounding each of these four edge motion events (Fig. 2C). Switching the sector width from 1 to 5 μm did not affect the patterns of the result maps, displaying robustness of the results over different window sizes (fig. S5). We found that the majority of Rac1-GTP and RhoA-GTP signals were located within 2 μm from the cell edge, while the highest traction force level was located 4 to 6 μm away from the cell edge, with a lower but correlating traction force at a depth of 1 to 2 μm (fig. S6). The universally low-level signals in the first window (0 to 1 μm away from the segmented cell edge) were most likely caused by segmentation imperfection. To avoid edge segmentation errors to cause artificial data fluctuations while entailing the most meaningful signals, we used the Rac1-GTP, RhoA-GTP, and traction force signals from the second sampling windows (1 to 2 μm away from the segmented cell edge) for further analysis (Fig. 3, A to E).

At the protrusion onset, high RhoA-GTP levels at the cell edge arose 20 to 30 s before an elevation of Rac1-GTP levels, in turn, occurring approximately 40 s before the protrusion onset that was paralleled by increased local traction force (Fig. 3A). High RhoA-GTP also appeared before the retraction onset, while traction force levels decreased before the retraction onset (Fig. 3A). These Rac1 and RhoA dynamics are consistent with previous studies (*7*, *9*, *11*, *12*, *14*, *16*, *32*, *34*) performed on glass/plastic surfaces with approximately million-fold higher stiffness, suggesting robustness of cell membrane dynamics regulation across environments with different mechanical properties.

Rac1-GTP levels gradually decreased after protrusion onset to reach a nadir at the protrusion *V*_max_ (Fig. 3A). Thus, a Rac1-GTP nadir at the protrusion *V*_max_ occurs at a restriction point, after which the membrane velocity slows down. Conversely, Rac1-GTP increased during retractions to reach a peak at the retraction *V*_max_ (Fig. 3A), suggesting that a Rac1-GTP peak occurs at the restriction point for cell membrane retractions.

In contrast to Rac1-GTP levels, RhoA levels gradually increased before the protrusion *V*_max_ and reached a peak 20 to 30 s after the protrusion *V*_max_. Before the retraction *V*_max_, RhoA levels began to decrease and reached a low 20 to 30 s after the retraction *V*_max_ (Fig. 3A). Around the protrusion and retraction *V*_max_, Rac1-GTP level alterations were also followed by RhoA-GTP level changes in the opposite direction (Fig. 3, A and D). RhoA-GTP and Rac1-GTP levels were inversely correlated around all four key membrane edge motion events (Fig. 3A), consistent with a RhoA-Rac1 inhibitory cross-talk (*1*, *9*, *11*, *34*–*36*).

Rac1-GTP levels displayed a positive correlation with traction force levels around each of these four membrane movement events with Rac1-GTP alterations preceding traction force changes by approximately 40 s (Fig. 3, A and E). Unexpectedly and contrary to Rac1-GTP, the local RhoA-GTP level fluctuations displayed a negative correlation with local traction force alterations around each of the four membrane movement events (Fig. 3, A and E).

**Fig. 4.**
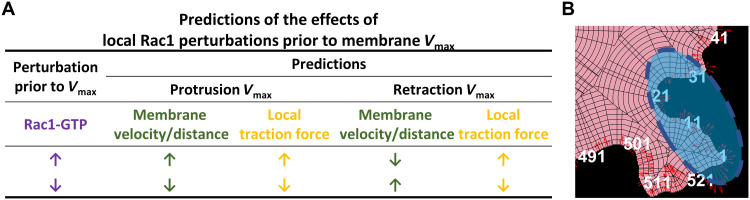
Predictions of the effects of local Rac1 perturbations. (**A**) Predictions of the effects of local Rac1 perturbations on cell membrane protrusions and retractions. The table indicates how activation (upward purple arrows) or inhibition (downward purple arrows) of Rac1-GTP before *V*_max_ would alter cell membrane velocity *V*_max_ and total membrane movement distance (green arrows), as well as the local traction force (yellow arrows). Arrow directions indicate predicted directions of change. Summary of the predictions: Local increase in Rac1-GTP levels before protrusion *V*_max_ will lead to later and higher protrusion *V*_max_ and longer protrusion distance. Inhibition of Rac1-GTP before protrusion *V*_max_ will lead to earlier and lower protrusion *V*_max_ and shorter protrusion distance. Increase in Rac1-GTP levels before retraction *V*_max_ will lead to earlier and lower retraction *V*_max_ and shorter retraction distance. Inhibition of Rac1-GTP levels before retraction *V*_max_ will lead to later and larger retraction *V*_max_ and longer retraction distance. Local increase in Rac1-GTP levels will increase the local traction force level in both protrusions and retractions, while inhibition of Rac1-GTP will decrease the local traction force levels in both protrusions and retractions. (**B**) Schematic image of optogenetic Rac1 perturbation setup. Local Rac1-GTP activity in a small region of the cells was elevated by constitutively active Rac1-GTP [PA-Rac1(Q61L)] or inhibited by dominant-negative Rac1-GDP [PA-Rac1(T17N)] with blue light. Cell membrane dynamics and traction force were imaged and quantified by confocal microscopy at 5-s intervals before, during, and after the perturbations.

**Fig. 5.**
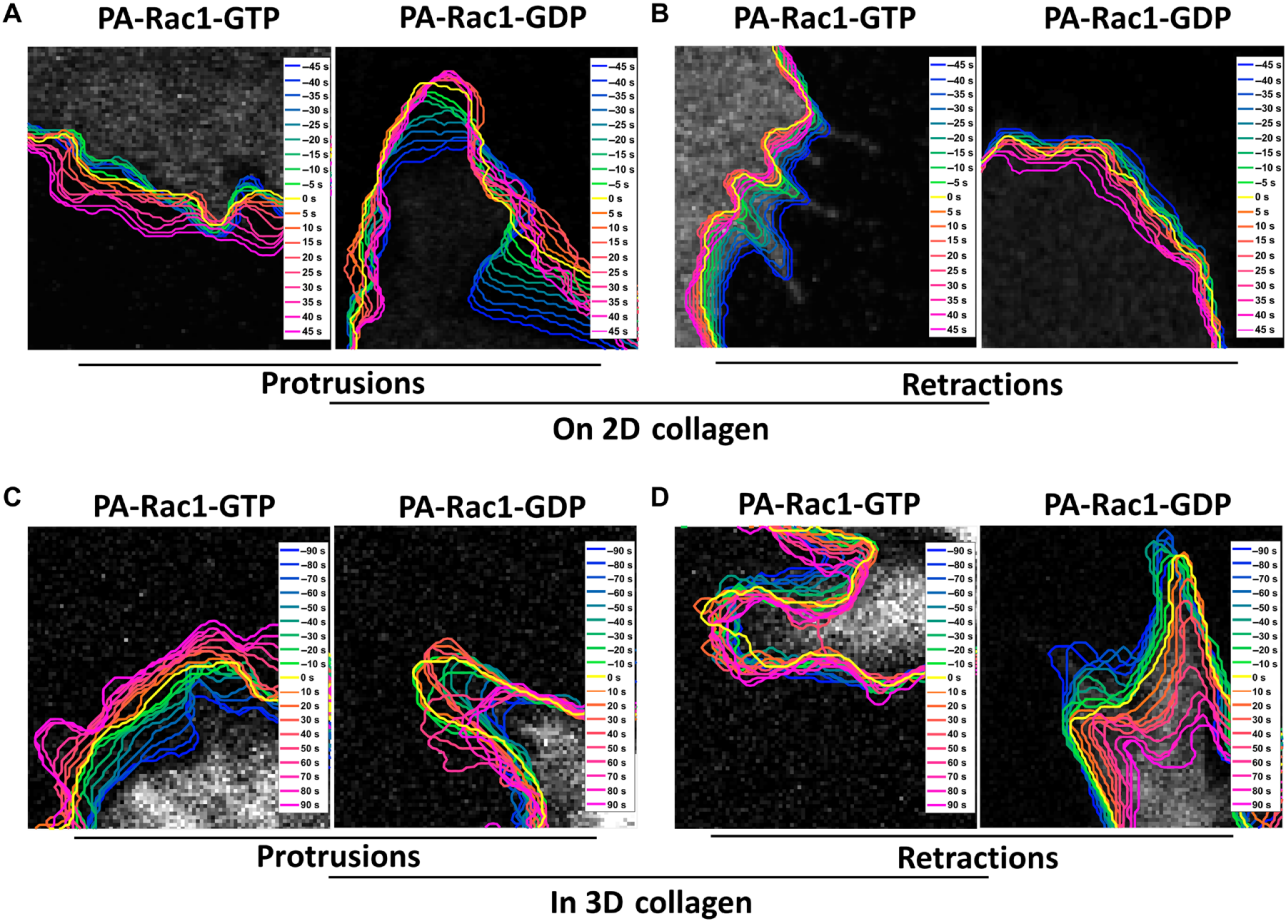
Local optogenetic Rac1 perturbations affect protrusions and retractions of HT1080 cells in 2D and 3D. (**A** and **B**) Sample time-lapse images of membrane protrusions and retractions upon Rac1 perturbations in HT1080 cells on collagen type I–coated PAA gel. Photoactivation of Rac1-GTP [PA-Rac1(Q61L)] or Rac1-GDP [PA-Rac1(T17N)] was performed in protruding (A) or retracting (B) cell areas. Cell images 45 s before photoactivation starts are shown in gray, as well as the following cell edge dynamics in pseudo-color coding for different time points as indicated. Yellow shows the photoactivation starting time point. Cold and warm colors show the cell edge location before and after the start of the photoactivation at the time points indicated in the insets. (**C** and **D**) Sample time-lapse images of membrane protrusions and retractions upon Rac1 perturbations in HT1080 cells embedded in 3D collagen. Photoactivation of Rac1-GTP [PA-Rac1(Q61L)] or Rac1-GDP [PA-Rac1(T17N)] was performed in protruding (C) or retracting (D) cell regions. Cell images 90 s before photoactivation starts are shown in gray, as well as the following cell edge dynamics in pseudo-color coding for different time points as indicated. Yellow shows the photoactivation starting time point. Cold and warm colors show the cell edge location before and after the start of the photoactivation at the time points indicated in the insets.

The local Rac1-GTP nadirs and peaks at the protrusion and retraction *V*_max_ made us hypothesize that Rac1-GTP levels may play a central role in restraining membrane protrusions and retractions. We therefore focused on Rac1 and postulated predictions that could be used for functional testing by available optogenetics-based local Rac1-GTP perturbations (Fig. 4). We predicted how local induction of Rac1-GTP or Rac1-GDP (inhibiting Rac1-GTP) before protrusion or retraction *V*_max_ would affect the velocity and the duration of protrusions and retractions, as well as its influence on local traction force (Fig. 4A). There is also the alternative possibility that the Rac1-GTP level changes are the consequence of the membrane dynamics rather than executing a regulatory role. This alternative would predict no membrane dynamic changes upon Rac1-GTP perturbation. According to our predictions in Fig. 4A, we designed and performed Rac1 perturbation experiments with genetically encoded optogenetic tools. Photoactivatable “constitutively active” Rac1-GTP [PA-Rac1(Q61L)] or “dominant-negative” Rac1-GDP [PA-Rac1(T17N)] (*37*) were induced by blue light in a small region of the HT1080 cells (Fig. 4B). As predicted by our hypothesis, activation of PA-Rac1-GTP enhanced membrane protrusions and restricted retractions, while activation of PA-Rac1-GDP restricted the ongoing protrusions and enhanced the retractions, both in 2D (Fig. 5, A and B) and in a 3D collagen gel (Fig. 5, C and D).

**Fig. 6.**
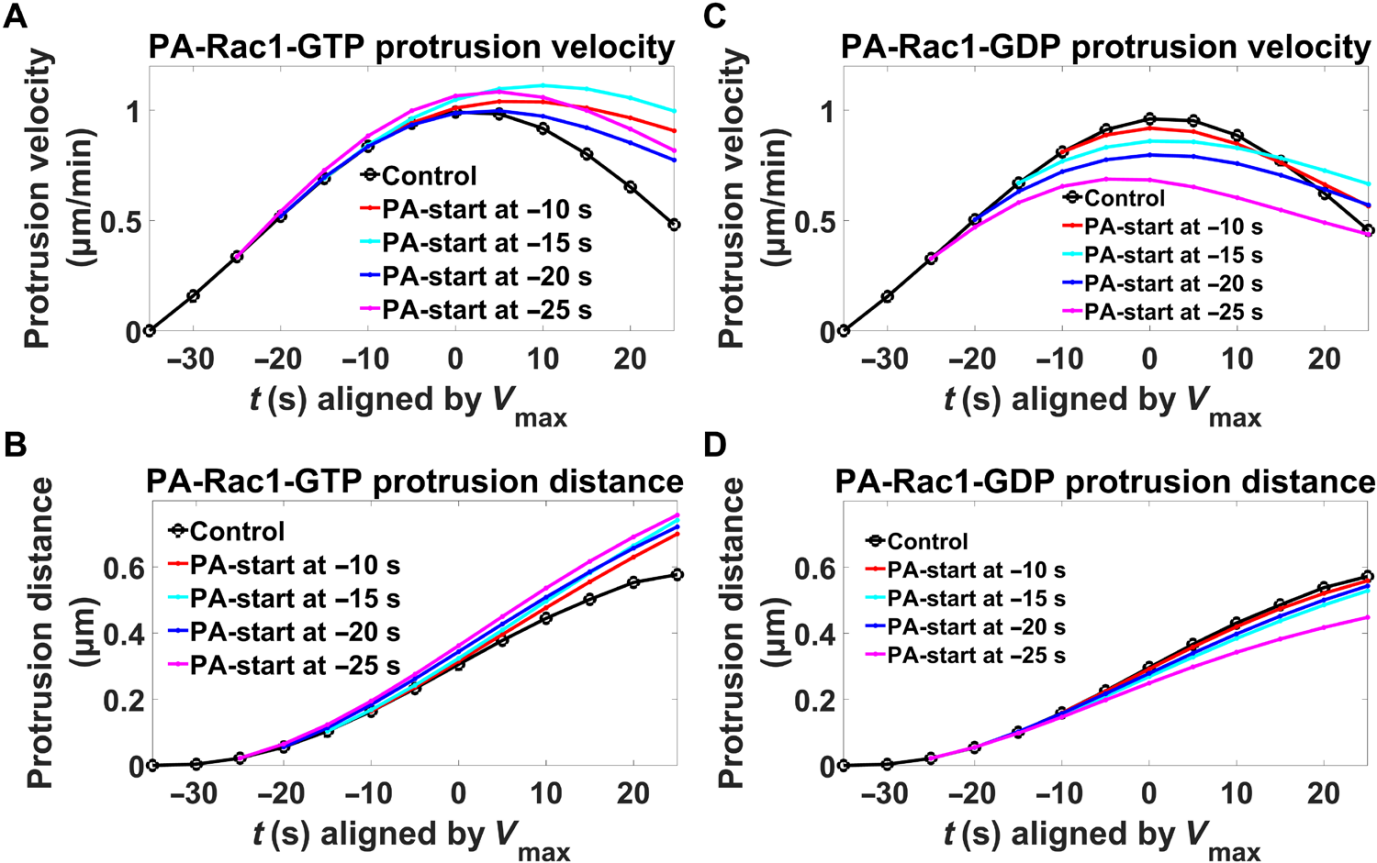
Role of local Rac1-GTP levels in restriction of protrusions. Mean membrane protrusion velocities (**A** and **C**) and distances (**B** and **D**) before and after *V*_max_ are plotted with the difference in difference (DID) method. Black lines, control (without photoactivation); colored lines, difference toward controls upon photoactivation. False discovery rate (FDR) analysis (*54*, *55*): Except for the −10 s PA-Rac1-GDP membrane protrusion distance, the *Q* values of all the other perturbations are smaller than the 0.1% threshold when compared to the corresponding controls. Sample details: *PA-Rac1-GTP*: control (*n* = 1545); −10 s [*n* = 97, velocity (*Q* = 5.05 × 10^−3^ and 0.1% threshold = 9.99 × 10^−1^) and distance (*Q* = 9.95 × 10^−1^ and 0.1% threshold = 9.99 × 10^−1^)]; −15 s [*n* = 86, velocity (*Q* = 1.38 × 10^−3^ and 0.1% threshold = 9.99 × 10^−1^) and distance (*Q* = 9.92 × 10^−1^ and 0.1% threshold = 9.99 × 10^−1^)]; −20 s [*n* = 82, velocity (*Q* = 9.81 × 10^−1^ and 0.1% threshold = 9.99 × 10^−1^) and distance (*Q* = 9.97 × 10^−1^ and 0.1% threshold = 9.99 × 10^−1^)]; and −25 s [*n* = 75, velocity (*Q* = 7.11 × 10^−1^ and 0.1% threshold = 9.99 × 10^−1^) and distance (*Q* = 9.95 × 10^−1^ and 0.1% threshold = 9.99 × 10^−1^)] from 83 cells of eight experiments; *PA-Rac1-GDP*: control (*n* = 1127); −10 s [*n* = 60, velocity (*Q* = 9.96 × 10^−1^ and 0.1% threshold = 9.99 × 10^−1^) and distance (*Q* = 9.99 × 10^−1^ and 0.1% threshold = 9.99 × 10^−1^)]; −15 s [*n* = 62, velocity (*Q* = 9.85 × 10^−1^ and 0.1% threshold = 9.99 × 10^−1^) and distance (*Q* = 9.997 × 10^−1^ and 0.1% threshold = 9.999 × 10^−1^)]; −20 s [*n* = 49, velocity (*Q* = 9.88 × 10^−1^ and 0.1% threshold = 9.99 × 10^−1^) and distance (*Q* = 9.997 × 10^−1^ and 0.1% threshold = 9.999 × 10^−1^)]; and −25 s [*n* = 73, velocity (*Q* = 8.33 × 10^−1^ and 0.1% threshold = 9.99 × 10^−1^) and distance (*Q* = 9.98 × 10^−1^ and 0.1% threshold = 9.99 × 10^−1^)] from 69 cells of eight experiments.

The window sampling method provided detailed quantitative results of the 2D derived time-lapse images, which allowed us to quantitatively compare the local membrane behavior differences before and after the local photoactivation. Induction of PA-Rac1-GTP before protrusion *V*_max_ extended the time until *V*_max_ was reached and also increased the *V*_max_ value. Consequently, the membrane protrusion distance was extended. The longer the time that the PA-Rac1-GTP was induced before the membrane protrusion *V*_max_, the stronger the protrusion elevation was (Fig. 6, A and B). On the contrary, induction of PA-Rac1-GDP before protrusion *V*_max_ led to earlier *V*_max_ arrival time, lower *V*_max_, and a shorter protrusion distance. In addition, in this case, the longer the time before protrusion *V*_max_ that PA-Rac1-GDP was induced, the earlier and smaller *V*_max_ and the shorter the protrusion distance were (Fig. 6, C and D). Thus, replacing the Rac1-GTP nadir occurring at the protrusion *V*_max_ with induction of Rac1-GTP caused prolonged protrusions, while enhancing the Rac1-GTP nadir through induction of Rac1-GDP further restricted the protrusions. This strongly indicates that the Rac1-GTP nadir functions to restrict protrusions.

**Fig. 7.**
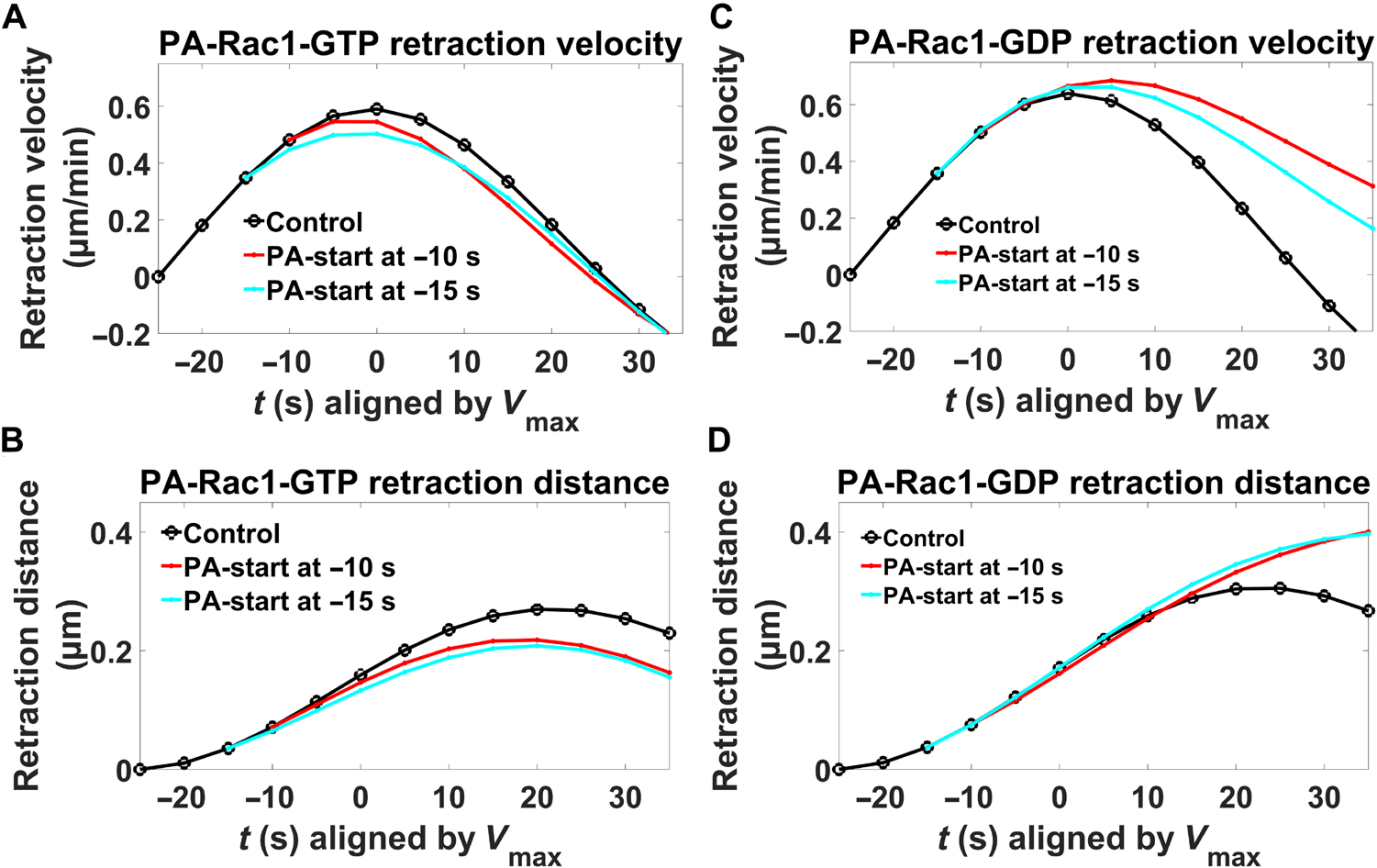
Role of local Rac1-GTP levels in restriction of retractions. Mean membrane retraction velocities (**A** and **C**) and distances (**B** and **D**) before and after *V*_max_ are plotted with the DID method. Black lines, control (without photoactivation); colored lines, difference toward controls upon photoactivation. FDR analysis (*54*, *55*): The *Q* values of all the perturbations in the graph are smaller than the 0.1% threshold when compared to the corresponding controls. Sample sizes: *PA-Rac1-GTP*: control (*n* = 162); −10 s [*n* = 52, velocity (*Q* = 9.987 × 10^−1^ and 0.1% threshold = 9.998 × 10^−1^) and distance (*Q* = 9.998 × 10^−1^ and 0.1% threshold = 9.999 × 10^−1^)]; and −15 s [*n* = 53, velocity (*Q* = 9.987 × 10^−1^ and 0.1% threshold = 9.998 × 10^−1^) and distance (*Q* = 9.997 × 10^−1^ and 0.1% threshold = 9.999 × 10^−1^)] from 83 cells of eight experiments; *PA-Rac1-GDP*: control (*n* = 120); −10 s [*n* = 60, velocity (*Q* = 1.81 × 10^−3^ and 0.1% threshold = 9.99 × 10^−1^) and distance (*Q* = 9.90 × 10^−1^ and 0.1% threshold = 9.99 × 10^−1^)]; and −15 s [*n* = 59, velocity (*Q* = 8.78 × 10^−2^ and 0.1% threshold = 9.99 × 10^−1^) and distance (*Q* = 9.94 × 10^−1^ and 0.1% threshold = 9.99 × 10^−1^)] from 69 cells of eight experiments.

Induction of PA-Rac1-GTP before retraction *V*_max_ shortened the time to *V*_max_, lowered the *V*_max_, and shortened the membrane retraction distance, all with a stronger effect the earlier the Rac1-GTP activation was added (Fig. 7, A and B). In contrast, local induction of PA-Rac1-GDP before retraction *V*_max_ delayed *V*_max_ occurrence, increased *V*_max_, and prolonged the retraction distance (Fig. 7, C and D). This means that enhancement of the Rac1-GTP peak occurring at retraction *V*_max_ further restricted the retractions, while counteracting this Rac1-GTP peak reversed the naturally occurring restriction. This indicates a regulatory role for the Rac1-GTP peak in the restriction of membrane retractions.

**Fig. 8.**
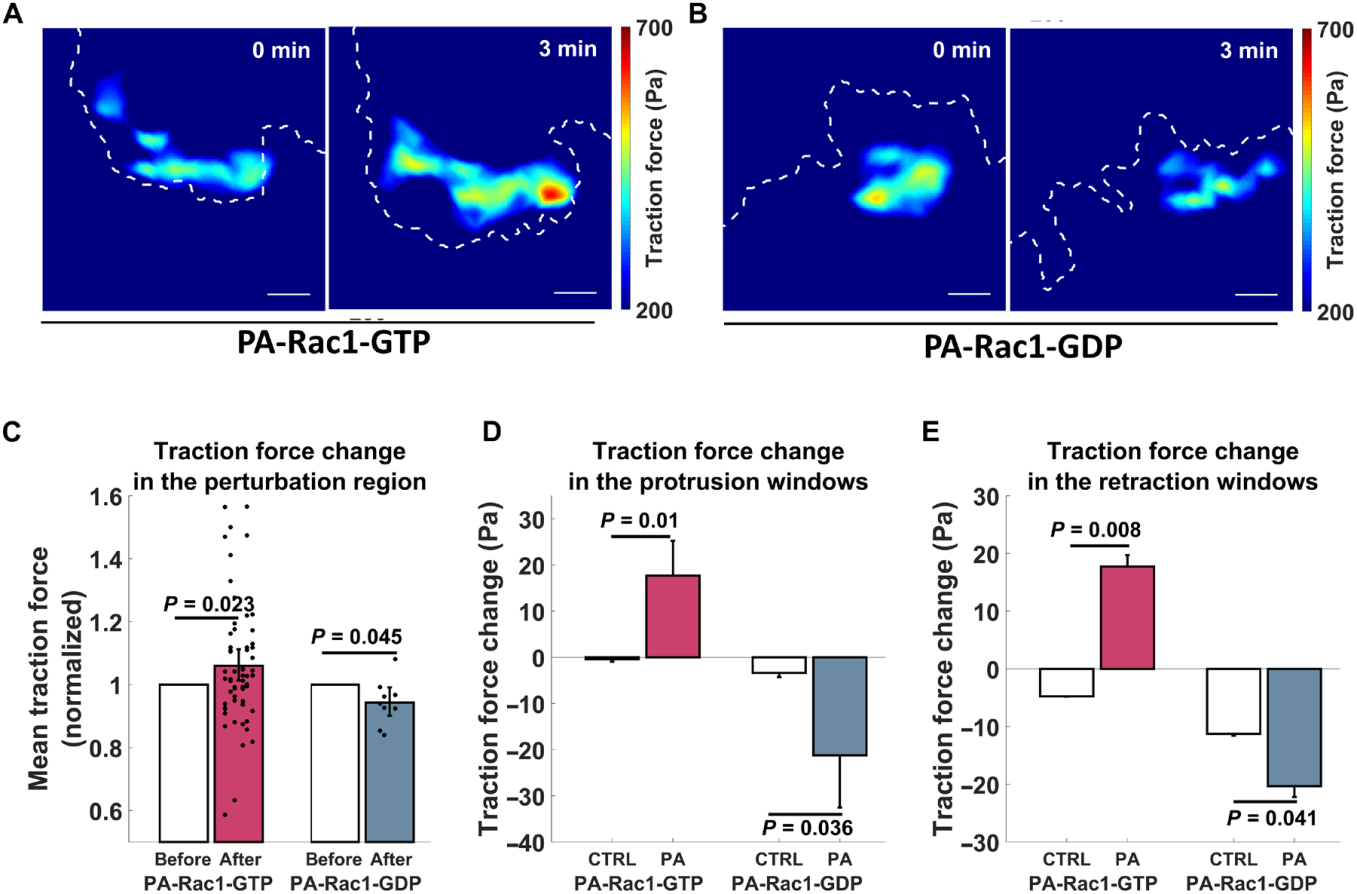
Local Rac1-GTP levels promote cell traction force. (**A** and **B**) Sample images of local traction force upon Rac1-GTP level perturbation. Local traction forces before and 3 min after photoactivation of Rac1-GTP [PA-Rac1(Q61L)] (A) or Rac1-GDP [PA-Rac1(T17N)] (B) are shown in pseudo-colors. Dashed white lines show the cell edge. All of these regions are within the photoactivation area. Scale bar, 5 μm. (**C** to **E**) Rac1-GTP levels affect local cell traction force. (C) Mean traction force in the photoactivation region 3 to 4 min after the start of induction of PA-Rac1-GTP or PA-Rac1-GDP compared to the mean traction force 0 to 1 min before induction. *P* values based on paired-sample *t* test. Results were derived from 60 cells (PA-Rac1-GTP) and 9 cells (PA-Rac1-GDP), respectively. (D to E) Mean traction force change in the second windows (1 to 2 μm from the segmented cell edge) of the corresponding protrusion (D) or retraction (E) sectors 2 min after the start of induction of PA-Rac1-GTP or PA-Rac1-GDP compared to the mean traction force change in the windows of the sectors before the perturbation started at time points when these sectors displayed corresponding phases of protrusions or retractions. *P* values based on two-sample *t* test. PA-Rac1-GTP results were derived from 9178 (control) or 641 (perturbed) protrusions and 925 (control) or 166 (perturbed) retractions of 83 cells of eight experiments. PA-Rac1-GDP results were derived from 6452 (control) or 385 (perturbed) protrusions and 619 (control) or 188 (perturbed) retractions of 69 cells of eight experiments.

Our finding of a consistent positive cross-correlation, where Rac1-GTP preceded traction force around the key membrane events (Fig. 3, A and E), suggests that Rac1-GTP may induce traction force. This suggestion was functionally validated since optogenetic activation of PA-Rac1-GTP increased traction force levels, while PA-Rac1-GDP inhibited the traction force within the cellular region exposed to optical PA-Rac1-GDP activation (Fig. 8, A to C). The window sampling-based quantification showed that, in both protrusion and retraction windows, induction of PA-Rac1-GTP increased the local traction force, while PA-Rac1-GDP decreased the local traction force (Fig. 8, D and E). Together, this defines a role for Rac1-GTP in the promotion of traction forces.

## DISCUSSION

We here define a broad role for Rac1 in the control of cell membrane dynamics, confirming its role in membrane protrusion initiation (*1*, *7*, *11*) and assigning previously unidentified functions for Rac1 in the restriction of both membrane protrusions and retractions. Thus, highly localized and temporally precise regulation of Rac1-GTP levels appears to be central for the dynamic membrane protrusion-retraction cycles that cells use to probe the microenvironment (*38*, *39*). Consistently, Rac appears critical for the probing behavior in *Dictyostelium* (*40*).

**Fig. 9.**
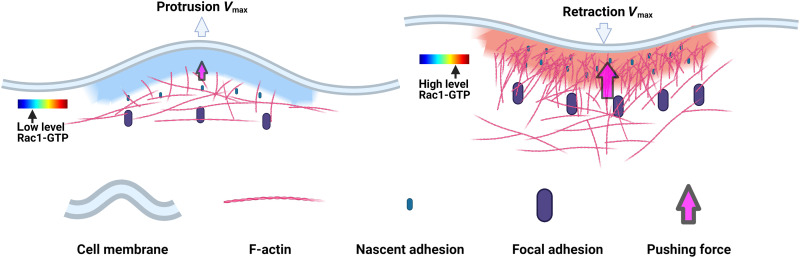
Schematic models for the role of Rac1 in restricting cell protrusions and retractions. Cell membrane protrusions and retractions are large transient structures used for probing of the microenvironment where the cell membrane locally protrudes or retracts usually followed by a reversion. After protrusion/retraction initiation, the local cell membrane velocity gradually increases until it reaches a peak (*V*_max_) that represents a restriction point, at which the protrusion/retraction is restrained and then reverted. We here found a role for Rac1-GTP activity in restricting membrane protrusions and retractions and that Rac1-GTP promotes local cellular traction forces. Left: We found that Rac1-GTP near the cell edge reaches high levels ~20 to 30 s before protrusion initiation. Then, Rac1-GTP levels gradually decline to reach a nadir at protrusion *V*_max_, a nadir that we found to functionally restrict protrusions. Low Rac1-GTP levels are linked to sparse non-networked membrane-proximal F-actin (*15*) and limited nascent adhesions (*8*). Given also that both F-actin meshwork density and cell-matrix adhesions provide forces supporting cell membrane protrusions, we hypothesize that the low Rac1-GTP at protrusion *V*_max_ causes reduced membrane support that restrains the local protrusion velocity. Right: We found that Rac1-GTP was low at the initiation of retractions and then gradually increased to peak at retraction *V*_max_, at which the Rac1-GTP peak restricts retractions. High Rac1-GTP is linked to a dense membrane-proximal F-actin meshwork (*15*), abundant nascent adhesions (*8*), and high cell traction force (Fig. 8, C to E). We therefore hypothesize that the high Rac1-GTP at retraction *V*_max_ results in abundant F-actin and cell-matrix adhesions that reduce the local retraction velocity by their supporting force to resist membrane retraction.

We conclude that local Rac1-GTP nadirs limit cell membrane protrusions. Low Rac1-GTP levels are linked to sparse non-networked membrane-proximal F-actin (*15*) and limited nascent adhesions (*8*). Given that both F-actin meshwork density and cell-matrix adhesions provide forces supporting cell membrane protrusions, we propose that the low Rac1-GTP at *V*_max_ may cause reduced membrane support that restrains the local protrusion velocity (Fig. 9, left). The Rac1-GTP peak-mediated restriction of cell membrane retractions may work in the opposite manner. High Rac1-GTP is linked to a dense membrane-proximal F-actin meshwork (*15*) and abundant nascent adhesions (*8*). We thereby infer that the high Rac1-GTP at retraction *V*_max_ may result in abundant F-actin and cell-matrix adhesions, which reduce the local retraction velocity by their strong supporting force to resist membrane retraction (Fig. 9, right). However, while Fam49 (family of unknown function 49)/CYRI (CYFIP-related Rac interactor)- and ARHGAP39 (Rho GTPase activating protein 39)–mediated Rac1 inhibition can inhibit protrusions and a large number of guanine nucleotide exchange factors, GTPase activating proteins, guanine nucleotide dissociation inhibitors, and the local GTP pool can control Rac1-GTP levels (*8*, *9*, *41*), it remains to be investigated how Rac1-GTP levels may be regulated to form local temporal nadirs and peaks during the heights of protrusions and retractions. Besides Rac1-GTP dynamics, other components may also contribute to the restriction of membrane movement. For example, we observed RhoA-GTP level changes around the protrusion and retraction *V*_max_, while other candidate components, such as Cdc42, the Wave and Arp2/3 complexes, F-actin, and actomyosin dynamics, were not analyzed here.

We report simultaneous FRET biosensor and traction force microscopy (TFM) measurements at submicrometer resolution, facilitated by applying the TFM beads at the surface of the PAA gel, compared to conventional TFM beads embedded within the gel that prevents concomitant FRET measurements. This way, we identified a time-lagged positive correlation between Rac1-GTP and traction force levels. By use of optogenetic Rac1 activation, we then defined a functional role of Rac1-GTP to promote traction forces. The function of Rac1 in promoting traction forces may therefore be brought about by the known function of Rac1 to promote F-actin networks and cell-matrix adhesions, both critical for the generation of traction force (*26*, *38*). This capability of Rac1 to promote traction force may contribute to the here identified role of Rac1 to restrict protrusions and retractions. Rac1 signaling may also alter the rearward F-actin transport across the lamellipodia corresponding to periodic lamellipodial contractions, which may create traction forces and direct cell migration (*42*–*44*). Observations and perturbations of focal complex formation, actomyosin contractility and polarization (*45*), and the Wave and Arp2/3 complexes (*46*), in combination with the measure of traction forces, membrane dynamics, and Rac1-GTP, all in the presence or absence of Rac1 perturbations, might provide mechanistic details about how the traction force and cell membrane dynamics are controlled by Rac1-GTP. In addition to HT1080 human fibrosarcoma cells, further studies will reveal whether Rac-GTP levels may also control the restriction of membrane protrusions and retractions in other organisms and cell types and during different types of cell migration. In summary, we found that local Rac1-GTP temporal fluctuations control the local membrane edge velocities that are critical for restricting the size and durability of protrusions and retractions.

## MATERIALS AND METHODS

### Cell line and culture

Human fibrosarcoma cell line HT1080 (gift from E. Sahai, Francis Crick Institute; before sending, the Sahai laboratory validated this cell line by comparing its STR (short tandem repeat) profile to the published ones) was cultured in Dulbecco’s modified Eagle’s medium (DMEM) (Gibco) and supplemented with 10% fetal bovine serum (FBS; Gibco), 1 mM sodium pyruvate (Gibco), penicillin (100 U ml^−1^), and streptomycin (100 μg ml^−1^; Gibco). Cells cultured less than 20 passage numbers were used in the experiments.

### Plasmids and stable cells

FRET biosensor containing vectors pTriEx4-Rac1-2G (Addgene plasmid no. 66110; http://n2t.net/addgene:66110; RRID (Research Resource identifiers): Addgene_66110) (*32*) and pTriExRhoA2G (Addgene plasmid no. 40176; http://n2t.net/addgene:40176; RRID: Addgene_40176) (*33*) were both gifts from O. Pertz. pmiRFP670-N1 was a gift from V. Verkhusha (Addgene plasmid no. 79987; http://n2t.net/addgene:79987; RRID: Addgene_79987) (*47*). The LifeAct-RFP670 construct was generated by digesting the pmiRFP670-N1 plasmid with Hind III and Age I restriction enzymes and then inserting the annealed LifeAct sequences (5′-agcttatgggcgtggccgacttgatcaagaagttcgagtccatctccaaggaggagcca-′3 and 5′-ccggtggctcctccttggagatggactcgaacttcttgatcaagtcggccacgcccata-′3) in between.

Stable cell lines expressing the biosensor constructs used for confocal microscopy were generated as follows. HT1080 cells were cotransfected with the Rac1-2G or RhoA-2G plasmids and the pGL4.21 vector containing a Puro^R^ cassette using Lipofectamine 2000 (Invitrogen) according to the manufacturer’s instructions. Forty-eight hours after transfection, cells were subjected to selection with puromycin (2 μg/ml; Sigma-Aldrich) and subsequently fluorescence-activated cell sorted (BD FACSAria) to obtain stably middle level–expressing cells.

### PAA gel preparation

PAA gel preparation on glass surface was adapted from previously published protocols (*30*) to enable simultaneous imaging of FRET biosensor and red fluorescent beads from the bottom (Fig. 1C). The details of the protocol for PAA gel preparation including all modifications from previous publications are provided below. NaOH (0.1 M) was added to the glass bottom of 35-mm MatTek dish for 5 min. The liquid was then removed, and the dish was air-dried. Approximately 150 μl of 3-aminopropyltrimethoxylsilane (Sigma-Aldrich) was added onto the NaOH-treated glass bottom from the previous step for 5 min. The dish was then washed thoroughly with ddH_2_O. Then, 2 ml of 0.5% glutaraldehyde in phosphate-buffered saline (PBS) was added to the MatTek dish for 30 min. The dish was subsequently thoroughly washed with ddH_2_O and air-dried. Another glass coverslip was sequentially coated with poly-d-lysine (0.1 mg ml^−1^) and 1:1000 ddH_2_O diluted red fluorescent beads (F8801, Invitrogen) for 30 min. Six microliters of an acrylamide/bis-acrylamide mixture dissolved in water (the concentrations of cross-linker and polymer were adjusted for a Young’s modulus of 6.9 kPa) (*29*), *N*-succinimidyl ester of acrylamidohexanoic acid (5.6 μg ml^−1^; N6 cross-linker) (*48*), 0.5% of ammonium persulfate, and 0.05% TEMED (tetramethylethylenediamine) were added to the center of the glass bottom of the previously treated MatTek dish. This gel mixture was covered with the red fluorescent bead–coated glass coverslip. To ensure that all red beads laid in the top plane of the gel, the dish was flipped during gelation. Once the polymerization was completed, the coverslip was removed carefully. Then, the gel was washed twice with PBS and coated with 2 ml of rat tail collagen type I (0.2 mg ml^−1^; Millipore) at 4°C overnight. Although a mixture of ECM proteins might provide an environment closer to the in vivo environment, we used a single component type I collagen as the substratum in our study to avoid the influence of local ECM differences to facilitate consistency within and across experiments.

### Confocal live-cell imaging

The collagen type I–coated PAA gel was washed with DMEM before usage. Cells were trypsinized, PBS-washed, and replated in DMEM (phenol red-free) + 1% FBS onto the gel in a MatTek dish (4000 cells in the inner well). Cells were then incubated at 37°C with 5% CO_2_ for 2 hours before imaging. Live-cell imaging was performed using a Nikon A1R confocal equipped with GaSaP (gallium arsenide phosphide) detectors and environmental chamber. Images were acquired every 10 s for 15 to 40 min, using a 60× Plan Apo oil objective [1.4 numerical aperture (NA)] and 1024 × 1024 resolution. FRET biosensor signals were acquired in: “mTFP1” channel (457-nm laser excitation and 482/35-nm emission) and “FRET” channel (457-nm laser excitation and 525/50-nm emission). Red fluorescent beads signal was acquired with 561-nm laser excitation and 595/50-nm emission. After the time-lapse imaging, cells were trypsinized and washed away. Then, the fluorescent beads in the same positions were imaged to capture the released state of the PAA gel for TFM reference. Cell migration velocity (between 75 and 90 μm/hour) was quantified by tracking and smoothing the center of area of the cells after the live-cell imaging.

### Lattice light-sheet microscopy

The preparation of PAA gel for lattice light-sheet microscopy (LLSM) was modified from the gel preparation on MatTek dish. The 5-mm round glass coverslip (Warner Instruments, catalog no. CS-5R) was surface-activated for gel conjugation, and the MatTek glass surface was coated with red fluorescent beads. In this way, the red fluorescent bead–labeled PAA gel was conjugated onto the 5-mm-diameter coverslip to fit for the LLSM imaging.

The LLSM used in these experiments is housed in the Advanced Imaging Center at the Howard Hughes Medical Institute, Janelia Research Campus. The system is configured and operated as previously described (*49*). Briefly, HT1080 cells were transiently transfected with Rac1 or RhoA biosensor and LifeAct-RFP670 plasmids (Fig. 1A) 16 to 24 hours before imaging using Lipofectamine 3000 (Invitrogen). Cells used for live-cell imaging were seeded on the collagen I–coated PAA gel conjugated on 5-mm round glass coverslip in Leibovitz L15 Medium, no phenol red (Thermo Fisher Scientific), containing 1% FBS. During imaging, cells were maintained at ~5% CO_2_ concentration, 95% humidity, and 37°C via custom-built environmental chamber (Okolab). Samples were illuminated by a 2D optical lattice generated by a spatial light modulator (Forth Dimension Displays). The light-sheet pattern was a square lattice with minimum NA of 0.44 and a maximum NA of 0.55. The sample is illuminated by 445-, 560-, and 642-nm diode lasers (MPB Communications) at 100, 10, and 90% AOTF (acousto-optic tunable filter) transmittance and 140-, 50-, and 100-mW initial box power through an excitation objective (0.65 NA, 3.74-mm WD; Special Optics). Fluorescent emission was collected by detection objective (CFI Apo LWD 25XW, 1.1 NA; Nikon) and a scientific complementary metal-oxide semiconductor camera (Hamamatsu Orca Flash 4.0 v2) at 75-ms exposure time. Acquired data were deskewed as previously described (*49*) and deconvolved using an iterative Richardson-Lucy algorithm. Point spread functions for deconvolution were experimentally measured using 200-nm TetraSpeck beads adhered to 5-mm glass coverslips (Invitrogen, catalog no. T7280) for each excitation wavelength.

### FRET ratio calculation

FRET image calculation was performed with Fiji (*50*). Images from mTFP1 channel and FRET channel were background-subtracted and smoothed with median filter. Then, the FRET/mTFP1 ratios were calculated and smoothed with median filter to get the final images. 2D median filter and 3D median filter (*51*) were used for confocal images and LLSM images, respectively.

### Traction force calculation

The traction force calculation was performed with the MATLAB-based software previously published by the Danuser Lab (*31*). Briefly, the fluorescent bead channel time-lapse images were registered toward the reference image to correct the stage drift. Then, in each time-lapse image, the displacement of the beads toward the reference image was calculated on the basis of which traction force was calculated with the Fourier transform traction cytometry method (*52*).

### Analysis of image series

The window sampling process was performed with the MATLAB-based software previously published by the Danuser Lab (*11*, *13*). Briefly, the automatic cell segmentation was performed on the basis of the images of the FRET channel. Then, the cell edge was sampled into 1-μm-wide sectors (or 5 μm for control purpose; fig. S5). For each sector, 1-μm-deep probing windows were created continuously from the cell edge to the cell center on the basis of its local geometry. The local edge sector velocity was obtained together with the mean Rac1-GTP or RhoA-GTP FRET signals and traction force level in its corresponding windows.

The quality of temporal alignment of the window sampled time-lapse images were further improved by comparing the traction force signals in the fifth sampling windows from neighboring time points in each cell. Then, the Rac1-GTP/RhoA-GTP and traction force signals from each window were smoothed over time with the MATLAB function csaps to suppress noise (Fig. 2B and fig. S7). Each time series of the mean GTPase activity and traction force levels obtained from a window provided one instantiation of the dynamics of Rac1-GTP or RhoA-GTP and traction force levels related to the corresponding cell edge sector motion.

The alignment of the Rac1-GTP/RhoA-GTP and traction force signals according to the corresponding edge sector protrusion/retraction onset/*V*_max_ was performed by following the previously published methods (*11*, *13*). Briefly, the protrusion and retraction time series of the edge sector were acquired on the basis of the local maxima/minima of the edge sector displacement. Protrusions/retractions with short distances (<1 μm) or time periods (<1 min) were discarded. Remaining protrusion/retraction time series were aligned according to the edge protrusion/retraction onset/*V*_max_ events and the mean values and 95% confidence intervals of the corresponding Rac1-GTP/RhoA-GTP, and traction forces around the four different events were calculated.

We focused most of our analysis on the windows located 1 to 2 μm from the segmented cell edge because we found them to most likely represent the actual cell edge (due to segmentation imperfection) and to entail the most meaningful dynamics of Rac1-GTP, RhoA-GTP, and traction forces (see Fig. 2C and fig. S5).

Although the absolute value is different, we found that the traction force in the second depth layer sampling windows were positively correlated to the traction force 4 to 6 μm away from cell edge with no time delay (fig. S6). Therefore, we also used signals in the second sampling windows for further analysis of traction force change dynamics, thereby also measuring and relating the RhoA-GTP or Rac1-GTP and traction forces in the exact same locations.

### Optogenetic perturbation

HT1080 cells stably expressing mCherry-tagged photoactivatable constitutively active (GTP-bound) Rac1 [PA-Rac1(Q61L)] or dominant-negative (GDP-bound) Rac1 [PA-Rac1(T17N)] (*37*) were seeded onto the collagen type I–coated PAA gel surface-labeled with far-red fluorescent beads. Two hours after cell seeding, local Rac1-GTP activity of a small region of the cells were elevated (PA-Rac1-GTP) for 1 min or inhibited (PA-Rac1-GDP) for 2 min with a pulsed 488-nm laser. Cell membrane dynamics (mCherry) and traction force (0.2-μm far-red beads; Invitrogen) images were obtained using an environmental chamber–equipped confocal microscope imaging at 5-s intervals from 5 min before the perturbation started until 5 min after the perturbation finished. After the time-lapse imaging, cells were detached from the PAA gel with trypsin, and reference images in the far-red channel were acquired for traction force calculation.

### Difference in difference method

The mean protrusion or retraction velocity/distance of the membrane edge sector time series in the perturbation region before photoactivation were extracted and aligned according to the onset time points as unperturbed controls. The mean time from protrusion or retraction onset until *V*_max_ reaching was obtained from these control protrusion or retraction velocity curves. The edge sectors that started to protrude or retract close to the perturbation starting time point were extracted and grouped according to the differences of their perturbation starting time and mean *V*_max_ reaching time. The mean velocities/distances of the perturbed protrusion or retraction time series in each group were calculated, and their differences toward corresponding control time series were then plotted.

### Quantification of traction force changes after optogenetic perturbation

To compare the traction force change with and without optogenetic perturbation in the photoactivated cell region, mean traction forces of each cell in the perturbed region before (0 to 1 min) and after (3 to 4 min) the perturbation starting time point were quantified. For each cell, the mean traction force after perturbation was normalized to the mean traction force before perturbation. Then, the normalized traction force changes after perturbation were compared after PA-Rac1-GTP or PA-Rac1-GDP induction.

To compare the traction force change after optogenetic perturbation in protrusion and retraction windows, cells were masked, and the windows were sampled according to the signal from the mCherry channel. Protrusion and retraction sectors starting near the perturbation starting time point were selected and aligned in the same way as described above. Control protrusion and retraction sectors were obtained on the basis of the time-lapse images before the perturbation started. Then, the mean traction force change 2 min after the start of induction of PA-Rac1-GTP or PA-Rac1-GDP in the second window (1 to 2 μm from the segmented cell edge) of the corresponding protrusion or retraction sectors initiated within 20 s before the perturbation started was compared to the mean traction force change in the windows of the sectors before the perturbation started at time points when these sectors displayed corresponding phases of protrusions or retractions.

### Optogenetic perturbation in 3D collagen

To prepare 3D cultures, 5 × 10^4^ HT1080 cells stably expressing mCherry-tagged photoactivatable constitutively active (GTP-bound) Rac1 [PA-Rac1(Q61L)] or dominant-negative (GDP-bound) Rac1 [PA-Rac1(T17N)] (*37*) were resuspended in 50 μl of serum-free culture medium and mixed with a solution containing 50 μl of 10× DMEM (Sigma-Aldrich), 50 μl of 0.26 M NaHCO_3_, 50 μl of H_2_O, 45 μl of 0.1 M NaOH, 5 μl of 200 mM glutamine, and 100 μl of collagen type I (6.7 mg/ml) from rat tail (Millipore). This solution was deposited in 96-well plates (100 μl per well). After polymerization at 37°C for 30 min, 100 μl of normal DMEM culture medium was added, and 3D cultures were incubated 24 hours before optogenetic perturbation. Local Rac1-GTP levels of a small region of the cells were elevated (PA-Rac1-GTP) or inhibited (PA-Rac1-GDP) for 2 min with a pulsed 488-nm laser. Cell membrane dynamics (mCherry) images were obtained using an environmental chamber equipped confocal microscope imaging at 5-s intervals from 5 min before the perturbation started until 5 min after the perturbation finished.

### Statistical analysis

The 95% bootstrap confidence intervals of the mean value of the aligned time series were calculated with bootstrap resampling method (MATLAB function bootci) (*53*). The false discovery rate (FDR) method (*54*, *55*) was performed with own MATLAB scripts. Because of the large difference of sample size between control (>1000) and perturbed (~50 to 100) groups, the same number of time series from the control group was randomly sampled toward the perturbed group 10,000 times. For each time, the SDs of the grouped time series (control and perturbed) were compared to the SD of the entire two-group time series with two sample *t* test for a *P* value at each time point. The grouped data were also randomly regrouped and SD compared for a control *P* value at each time point. After the 10,000 time comparisons, the *Q* values of positive FDR threshold was calculated [MATLAB function mafdr()] on the basis of the *P* values derived from both grouped data and randomized data. The 0.1% threshold of *Q* value was defined from the randomized data, and the mean *Q* value from the grouped data was compared to the threshold for significance. The paired *t* test was performed with MATLAB [function ttest()], and the two-sample *t* test was performed with MATLAB [function ttest2()].

## Notes

View/request a protocol for this paper from *Bio-protocol*.
