# Supplementary material for: Local temporal Rac1-GTP nadirs and peaks restrict cell protrusions and retractions
Source: Sci Adv. 2022 Mar 23;8(12):eabl3667. doi: 10.1126/sciadv.abl3667 (PMC8942371; doi:10.1126/sciadv.abl3667)
Supplement: Supplementary file 1 — Figs. S1 to S7 [file sciadv.abl3667_sm.pdf]

Science Advances
AAAS

Supplementary Materials for

Local temporal Rac1-GTP nadirs and peaks restrict cell protrusions and retractions

Jianjiang Hu*, Xiaowei Gong, Staffan Strömblad*

*Corresponding author. Email: staffan.stromblad@ki.se (S.S.); jianjiang.hu@ki.se (J.H.)

Published 23 March 2022, *Sci. Adv.* **8**, eabl3667 (2022)
DOI: 10.1126/sciadv.abl3667

The PDF file includes:

Figs. S1 to S7
Legend for movie S1

Other Supplementary Material for this manuscript includes the following:

Movie S1

Fig. S1

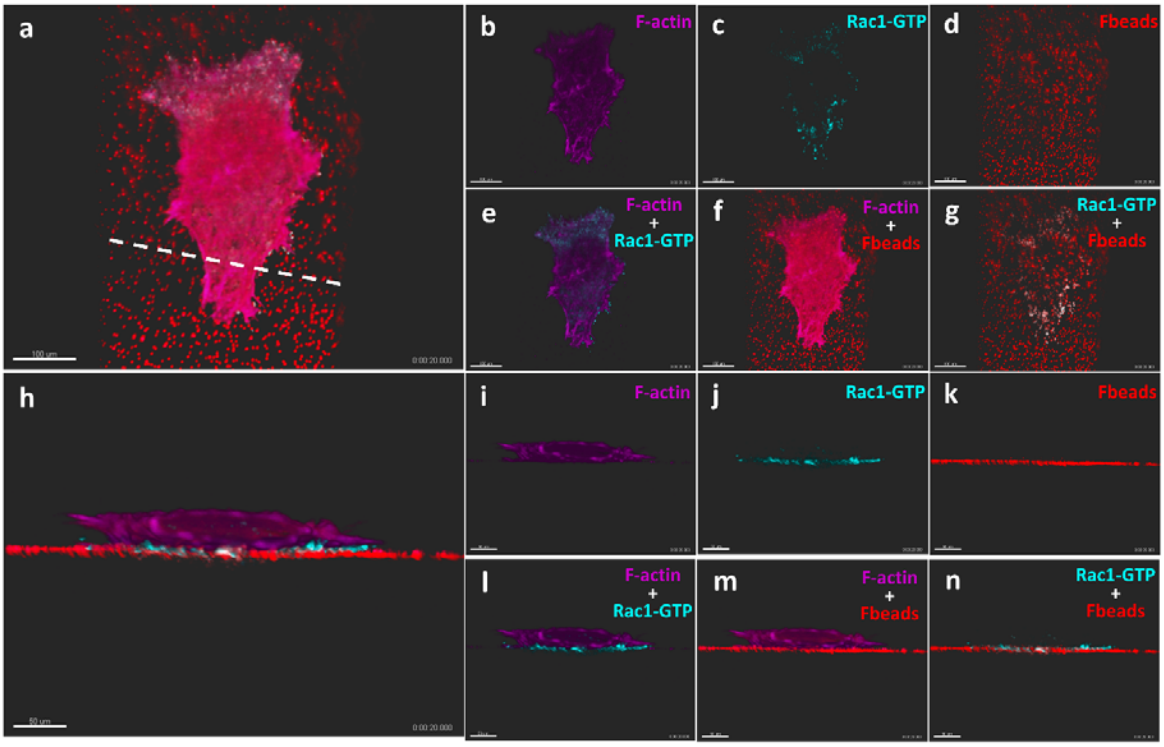

Fig. S1. Lattice light sheet microscopy displays Rac1-GTP at the cell-matrix interface. HT1080 cells expressing a Rac1-GTP biosensor and miRFP670 tagged F-actin were grown on a Collagen type I coated PAA gel surface labeled with red fluorescent beads. Rac1-GTP levels (cyan), fluorescent beads (red) and F-actin (purple) were imaged in 3D in live cells by lattice light sheet microscopy. Upper images (**a-g**): xy view of a sample cell, scale bar 100 µm. Lower images (**h-n**): side view of the sample cell via the white dash line in **a**, scale bar 50 µm. Left large images (**a, h**) show the composition of all three channels. Small images on the right show the single channels (**b-d, i-k**) and composed images of two channels at the time (**e-g, l-n**).

Fig. S2

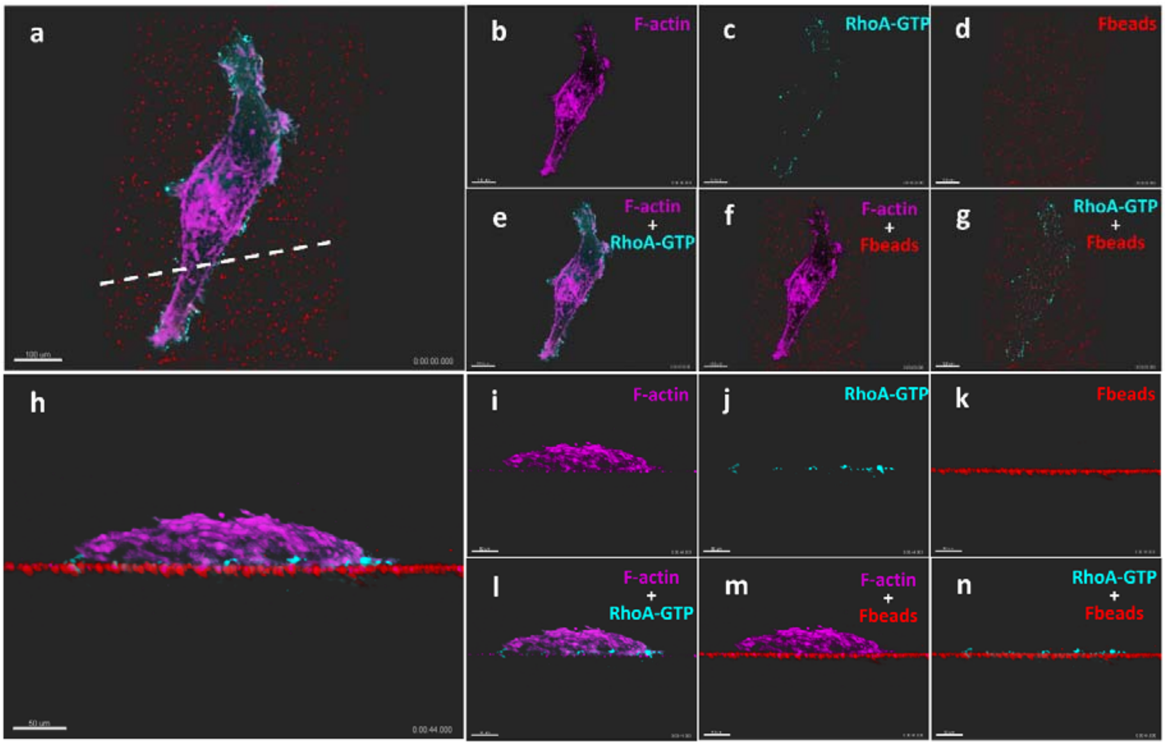

Fig. S2. Lattice light sheet microscopy displays RhoA-GTP at the cell-matrix interface. HT1080 cells expressing RhoA biosensor and miRFP670 tagged F-actin were grown on a Collagen type I coated PAA gel surface-labeled with red fluorescent beads. RhoA-GTP levels (cyan), fluorescent beads (red) and F-actin (purple) were imaged in 3D in live cells by lattice light sheet microscopy. Upper images (**a-g**): xy view of a sample cell, scale bar 100 µm. Lower images (**h-n**): side view of the sample cell via the white dash line in **a**, scale bar 50 µm. Left large images (**a, h**) show the composition of all three channels. Small images on the right show the single channels (**b-d, i-k**) and composed images of two channels at the time (**e-g, l-n**).

Fig. S3

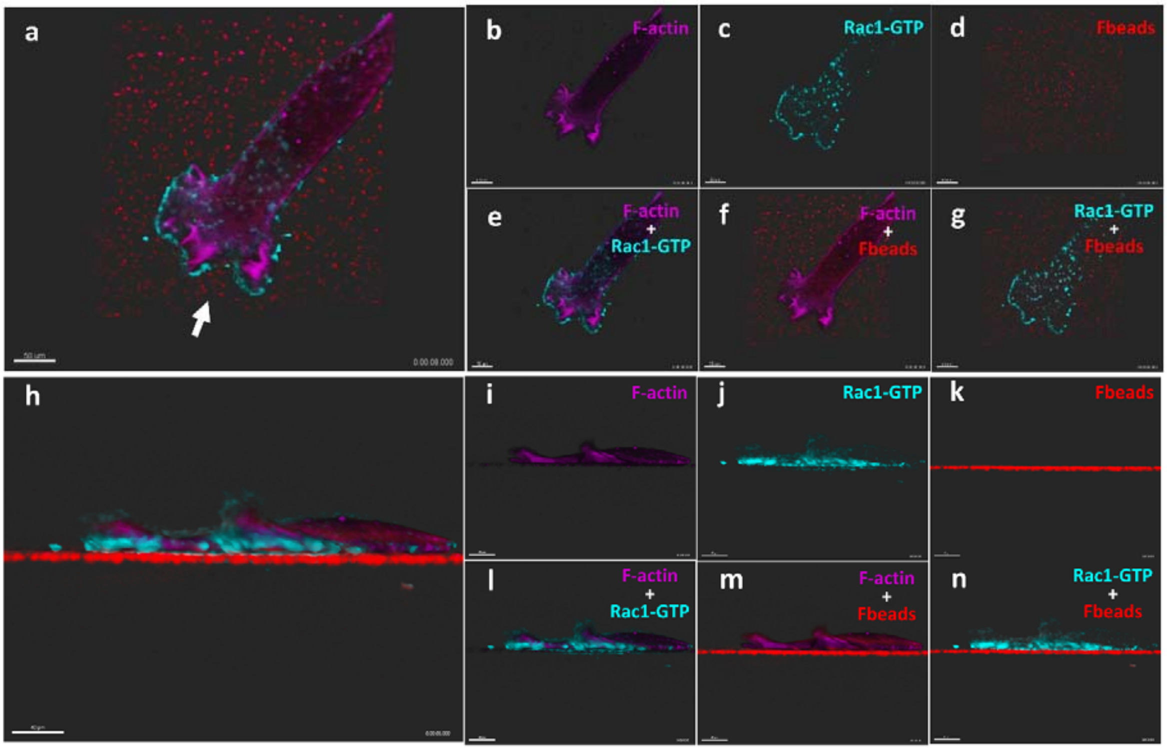

Fig. S3. Lattice light sheet microscopy displays low levels of Rac1-GTP at membrane ruffles.

HT1080 cells expressing Rac1 biosensor and miRFP670 tagged LifeAct were grown on a Collagen type I coated PAA gel surface-labeled with red fluorescent beads. Rac1-GTP levels (cyan), fluorescent beads (red) and F-actin (purple) were imaged in 3D in live cells. Upper images (**a-g**): xy view of a sample cell with a protrusion membrane ruffle, scale bar 50 µm. Lower images (**h-n**): side view of the sample cell via the white arrow in **a** that points to a protrusion ruffle above the cell-ECM interface, scale bar 40 µm. Left large images (**a, h**) show the composition of all three channels. Small images on the right show the single channels (**b-d, i-k**) and composed images of two channels at the time (**e-g, l-n**).

Fig. S4

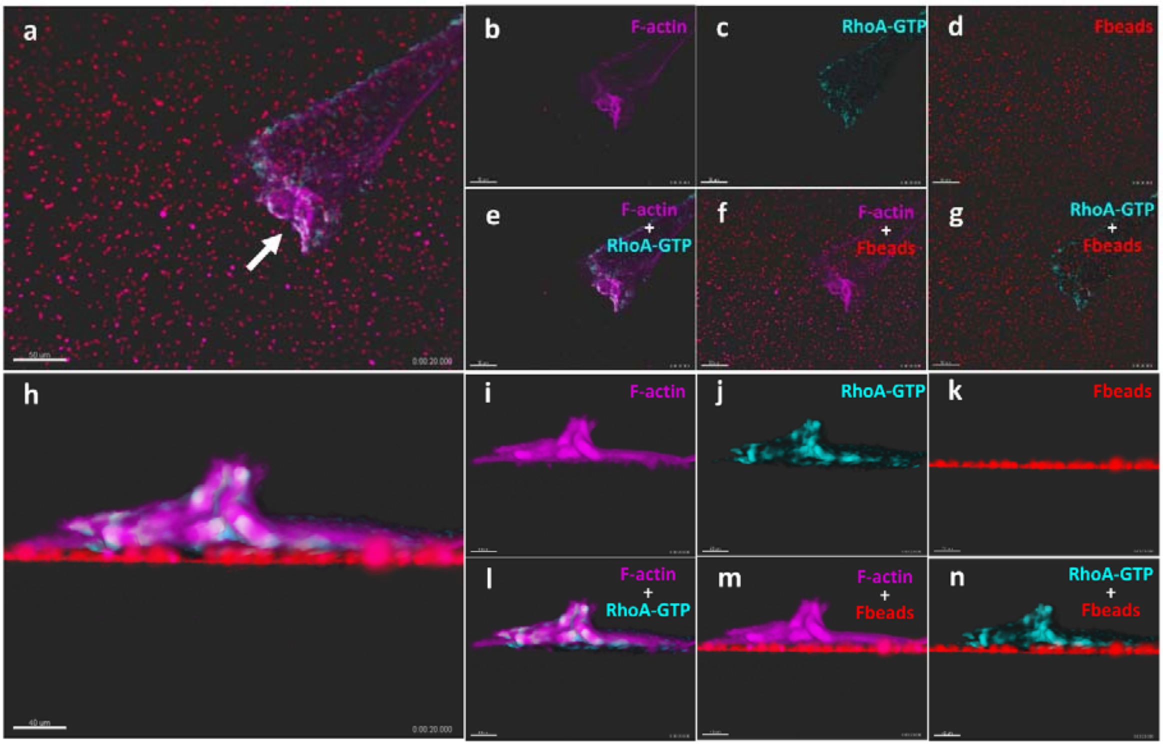

Fig. S4. Lattice light sheet microscopy displays high levels of RhoA-GTP at membrane ruffles.

HT1080 cells expressing RhoA biosensor and miRFP670 tagged LifeAct were grown on a Collagen type I coated PAA gel surface-labeled with red fluorescent beads. RhoA-GTP levels (cyan), fluorescent beads (red) and F-actin (purple) were imaged in 3D in live cells. Upper images (**a-g**): xy view of a sample cell with a protrusion membrane ruffle, scale bar 50 µm. Lower images (**h-n**): side view of the sample cell via the white arrow in **a** that points to a protrusion ruffle with high RhoA-GTP levels above the cell-ECM interface, scale bar 40 µm. Left large images (**a, h**) show the composition of all three channels. Small images on the right show the single channels (**b-d, i-k**) and composed images of two channels at the time (**e-g, l-n**).

Fig. S5

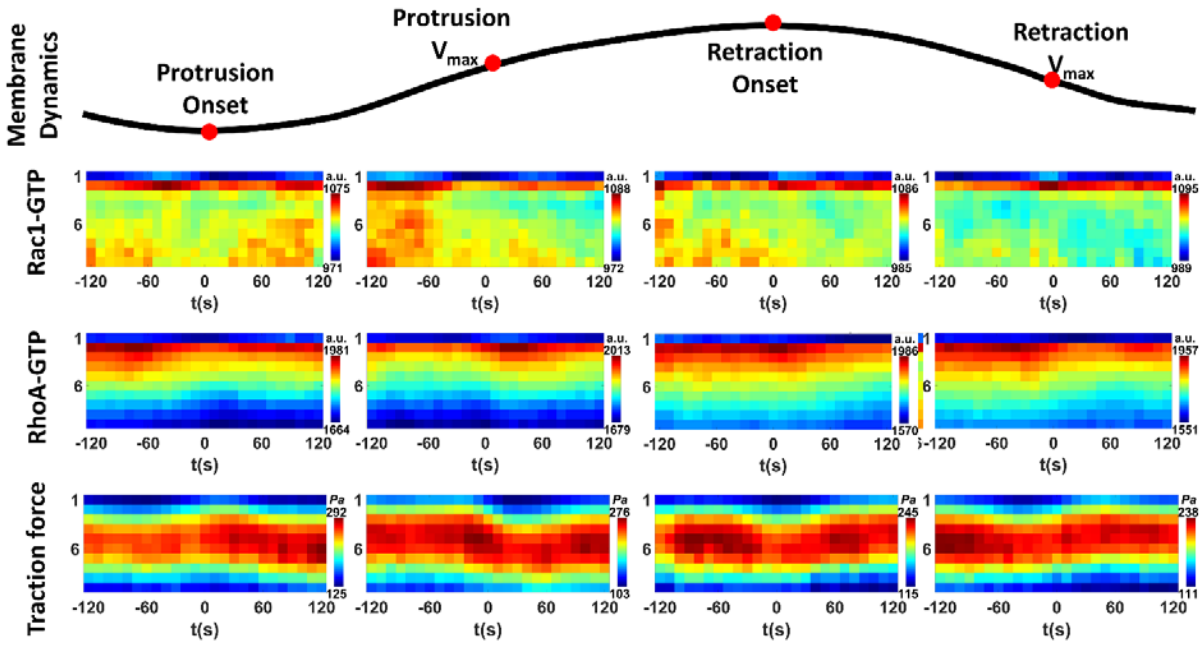

Fig. S5. Rac1-GTP, RhoA-GTP and traction force levels around key cell membrane events analyzed with an alternative sample window size.

The dynamics of 5 µm wide segmented cell membrane edge sectors were aligned according to cell membrane protrusion onset, maximal protrusion velocity (protrusion V_{max}), retraction onset, and maximal retraction velocity (retraction V_{max}). The mean values of Rac1-GTP, RhoA-GTP and traction force dynamics at different depths of the cell (1-10 µm, y axis) around the specific time points (-120 s to + 120 s, x axis) are shown in pseudo-color. Sample size: Rac1-GTP & traction force: 1056 protrusions and 796 retractions from 13 cells; RhoA-GTP: 1631 protrusions and 1023 retractions from 23 cells. The outcome is almost identical with that using 1 µm wide sector (Fig. 1d) showing that the results are insensitive to the segmentation sector width up to 5 µm.

Fig. S6

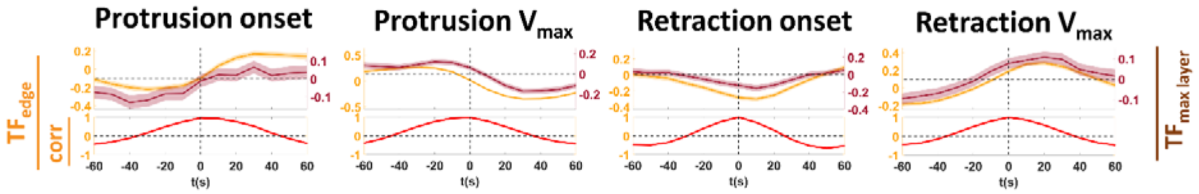

Fig. S6. High correlation of traction force dynamics at different cell depth windows.

The highest traction force levels occurred 4-6 um away from the cell edge. Here we show cross-correlation analysis of mean traction force levels in the second depth window (TF_{edge}, 1-2 µm from the segmented cell edge) with the corresponding mean traction force levels in the 4th and 5th depth layers of the window ($TF_{max\ layer}$, 4-6 µm away from cell edge) during protrusion onset, protrusion V_{max}, retraction onset and retraction V_{max}. For the above paired data, Solid lines show the mean values and the shadows show the 95% confidence interval. Correlation results were shown in red lines below the corresponding pairs.

Fig. S7

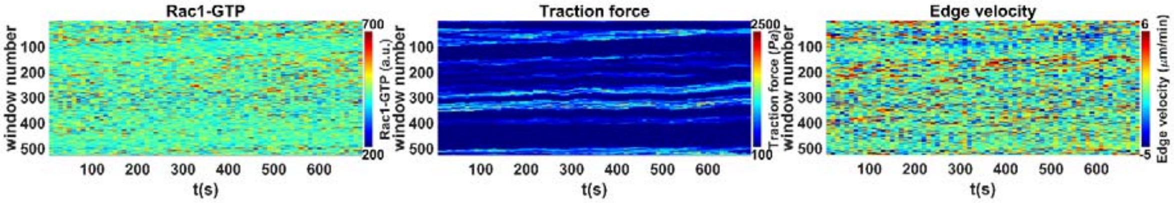

Fig. S7. Kymographs of GTPase and traction force levels and the corresponding edge velocity before alignment and smoothing.

Temporal dynamics of Rac1-GTP levels (left) and traction force (middle) levels in the second depth windows (1-2 µm from the cell edge) of a cell, as well as corresponding edge velocity (right) of each sector without alignment and smoothing. All graphs display pseudo colors as indicated in the insets. Corresponds to registered and smoothed graphs in Fig 1c.

Movie S1. Time lapse movies corresponding to images in Figure 2a.

The sample time lapse movies show the measured Rac1-GTP level (a), traction force (b), signals in the yfp channel (c) and outlay of the window sampling results (d) during cell migration. Rac1-GTP (a.u.) and traction force (*Pa*) levels are shown in pseudo colors according to the inset scales. Signals in the yfp channel indicate the distribution of Rac1 biosensor in the cell. For the window sampling images, the pink area shows the segmented cell area and the black lines show how the cells are sampled into 1 μm wide cell edge windows based on local cell edge geometry. Windows are numbered in white along the cell edge around the cell. Red arrows show the instant edge sector velocity. Scale bar 20 μm.
